# Signs of dysregulated fibrinolysis precede the development of type 2 diabetes mellitus in a population-based study

**DOI:** 10.1186/1475-2840-11-152

**Published:** 2012-12-18

**Authors:** Jenny Hernestål-Boman, Margareta Norberg, Jan-Hakan Jansson, Mats Eliasson, Jan W Eriksson, Bernt Lindahl, Lars Johansson

**Affiliations:** 1 Department of Public Health and Clinical Medicine, Umeå University, Umeå, Sweden; 2 Department of Medicine, Sunderby Hospital, Luleå, Sweden; 3 Department of Molecular and Clinical Medicine, Sahlgrenska University Hospital, Gothenburg and AstraZeneca R&D, Mölndal, Sweden

**Keywords:** Diabetes mellitus type 2, Tissue plasminogen activator, Plasminogen activator inhibitor-1, Von Willebrand factor, Fibrinolysis, Population study, Västerbotten Intervention Programme

## Abstract

**Background:**

Diabetic patients experience stimulated coagulation and dysfibrinolysis, which is associated with an increased risk of cardiovascular events. This imbalance may precede the manifest diagnosis. We investigated whether elevated antigen levels of tissue plasminogen activator (tPA), plasminogen activator inhibitor-1 (PAI-1), the tPA/PAI-1 complex, or von Willebrand Factor (VWF) precede type 2 diabetes mellitus (T2DM) diagnosis, and whether this elevation occurs before increased fasting plasma glucose (FPG) or 2-hour plasma glucose (2hPG) in individuals who later develop T2DM.

**Methods:**

We conducted a prospective incident case-referent study within the Västerbotten Intervention Programme. Cardiovascular risk factor data as well as FPG and 2hPG and blood samples for future research were collected at a baseline health examination between 1989 and 2000, (n= 28 736). During follow-up in January 2001, 157 cases had developed T2DM. Referents without T2DM were matched for sex, age, and year of participation (n=277). Subgroup analysis was performed for cases with normal baseline glucose levels (FPG <6.1 mmol/L and 2hPG < 8.9 mmol/L) and cases with elevated levels (FPG 6.1-6.9 mmol/L and/or 2hPG 8.9-12.1 mmol/L).

**Results:**

After adjusting for BMI, family history of diabetes, physical activity, smoking, systolic blood pressure and levels of C-reactive protein and triglycerides, independent associations were found between incident T2DM and elevated levels of tPA (OR=1.54, 95% CI 1.06-2.23), PAI-1 (OR=1.61, 95% CI 1.14-2.28), and tPA/PAI-1 complex (OR=2.45, 95% CI 1.56-3.84). In participants with normal glucose levels, PAI-1 (OR=2.06, 95% CI 1.10 - 3.86) exhibited an independent relationship with incident T2DM after the adjustments.

**Conclusions:**

Elevated levels of fibrinolytic variables precede the manifestation of T2DM after adjusting for metabolic and cardiovascular risk factors and can be detected several years before changes in glucose tolerance.

## Background

Smoking [1], low physical activity [2], obesity, insulin resistance, and hyperglycaemia [3] are independently associated with incident type 2 diabetes mellitus (T2DM). Insulin resistance is affected by metabolic risk factors for both T2DM and cardiovascular disease, including overall obesity, central obesity, elevated triglyceride levels, low HDL levels, hyperglycaemia, and hypertension [4]. Hyperglycaemia has been shown to stimulate coagulation in healthy humans, and hyperinsulinaemia has been associated with impaired fibrinolysis [5]. Diabetic patients have elevated levels of coagulation factors and impaired fibrinolysis, inducing a hypercoagulable state that may contribute to the increased risk of atherothrombotic events and venous thromboembolism [6-9]. Although reducing fasting plasma glucose levels to normal is seen as a way to prevent negative cardiovascular outcomes, such as myocardial infarction or stroke [10], the most recent study with insulin-treated T2DM patients failed to prove any protective effect of tight glycaemic control [11].

High levels of tPA have been shown to be predictive of future T2DM independent from metabolic syndrome [12,13]. Another study showed that tPA and PAI-1 levels are similar in patients with newly diagnosed T2DM compared to patients with T2DM for a long duration [14]. These findings indicate that impaired fibrinolysis in diabetic patients precedes the manifest diagnosis.

Some investigations have demonstrated that PAI-1 is predictive of T2DM but that its predictive ability disappears after adjusting for markers of metabolic syndrome [15,16]. This observation suggests that high plasma PAI-1 levels are associated with factors involved in metabolic syndrome, mainly obesity [17]. Other studies have indicated that high baseline PAI-1 levels are associated with incident diabetes [18] and that PAI-1 levels continue to increase with increasing glucose levels and the development of T2DM [19,20]. The tPA/PAI-1 complex, tPA bound to PAI-1, has been associated with cardiovascular disease [21,22], but its relation to incident diabetes is unknown.

Elevated von Willebrand Factor (VWF) levels increase the risk of cardiovascular events in patients with T2DM [23] but have not been shown to be associated with incident T2DM [13]. VWF is produced by endothelial cells, which may be activated by proinflammatory cytokines [24] such as IL-6, which in turn have been associated with an increased risk of incident T2DM [25]. C-reactive protein (CRP) has also been associated with an increased risk of incident T2DM, though this association was lost after adjusting for IL-6 [13].

To investigate the relationship between haemostatic factors and incident T2DM, we used a prospective population-based case-referent study. Our primary aim was to investigate whether elevated antigen levels of tPA, PAI-1, tPA/PAI-1 complex, and vWF precede the diagnosis of T2DM. Our secondary aim was to study whether these haemostatic variables increase prior to increased FPG or 2hPG levels in individuals that later develop T2DM.

## Methods

### Study population

**Figure 1:**
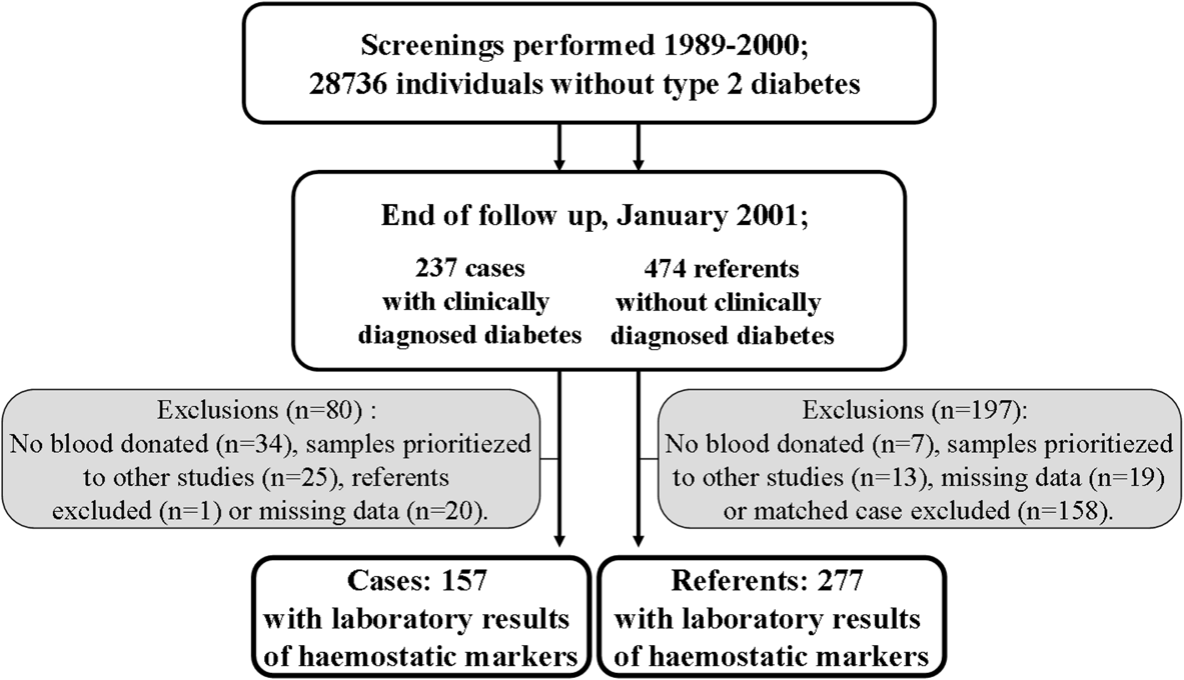
Schematic figure of study population.

We conducted a nested case-referent (case–control) study within the Västerbotten Intervention Programme (VIP) [26], a cardiovascular disease intervention project (Figure 1). All patients in Västerbotten County in northern Sweden were invited to participate in a health survey at their local primary-care centre when they were 30, 40, 50, or 60 years of age. At the health examination, data about age, sex, and cardiovascular risk factors were collected. Participants were also asked to donate a blood sample for research, which was stored at the Northern Sweden Medical Research Bank. The participation rate was high; the total study population from 1989 to 2000 represented 52% of the eligible population [27]. The vast majority of the participants were Caucasian. Between 1989 and 2000 the number of VIP participants in the Umeå region without diabetes type 1 or 2 at the time of their health examination was 28 736. A study of selection bias showed small differences between participants and non-participants [28].

Of all VIP participants examined between 1989 and 2000, 237 were diagnosed with T2DM after their examination and before January 2001 (Figure 1). T2DM was diagnosed according to World Health Organisation (WHO) criteria 1999 [29]. Participants were excluded if fasting plasma glucose (FPG) levels exceeded 6.9 mmol/L or 2-hour plasma glucose (2hPG) levels exceeded 12.1 mmol/L (n=11), if the participants had chosen not to donate blood (n=34), if the samples were prioritized to other studies (n=25), if data were missing (n=8), or if there were no matching referents (n=2). Two referents with stored plasma samples and without T2DM according to the registry were randomly selected for each case and matched for sex, age, and year of health examination. Thus, we included 157 cases with T2DM diagnosis during follow-up and 277 referents.

### Measurements

Body mass index (BMI) was calculated as measured weight in kilograms divided by the square of the height in metres (kg/m^2^). Smoking was categorized into three groups: non-smokers, ex-smokers, or daily smoking. Participants who reported that they smoked irregularly were categorised as ex-smokers. Participants were considered to have a family history of diabetes if they reported having a parent or sibling with T2DM. Physical activity was categorised into three levels based on the questionnaire about self-reported leisure time exercise and commuting habits during winter, as physical activity during the rest of the year was presumed to be at least as high as the activity during winter: 1) sedentary, never exercise, cycle and/or walk during their leisure time less than 2–3 times per month, take bus or car to work, or cycle and/or walk to work less than 2 km; 2) moderate, exercise now and then but not regularly or at most once a week, cycle and/or walk during their leisure time at least 2–3 times per week, or cycle and/or walk to work 2–5 km; 3) active, moderately active person who trains at least 2–3 times/week, or cycle or walk to work more than 5 km.

Hypertension was defined as systolic blood pressure ≥ 140 mmHg and/or diastolic blood pressure ≥ 90 mmHg and/or reported use of antihypertensive medication during the 14 days prior to the health examination. Blood pressure was measured with a mercury sphygmomanometer with the participant in the supine position after a 5-min rest.

Participants were instructed to fast from midnight until the time of blood sampling. Venous blood samples were drawn with minimum stasis in a sitting position into evacuated glass tubes containing 1/100 volume of 0.5% EDTA. The sample tube was centrifuged at 1500 x *g*; the plasma was immediately frozen at −20°C and stored at −80°C in the Northern Sweden Medical Research Bank until analysis. Oral glucose tolerance tests (OGTTs) were performed with a 75-g glucose load according to WHO standards. Fasting glucose concentrations were measured in venous plasma and glucose concentrations 2 hours after glucose intake in capillary plasma on a Reflotron bench-top analyser (Boehringer Mannheim GmbH, Mannheim, Germany). Triglycerides were analysed in thawed frozen samples via routine methods at the Department of Clinical Chemistry at Umeå University Hospital. ELISA reagent kits for tPA antigen, PAI-1 antigen, and the tPA/PAI-1 complex were purchased from Biopool (Umeå, Sweden). The inter-assay coefficients of variation (CVs) were: tPA 10.8%, PAI-1 7.1%, and tPA/PAI-1 complex 8.3%. Reagent kits with identical batch numbers were used for each analysis. The VWF levels were analysed with reagents purchased from DAKO (Copenhagen, Denmark). The CV for VWF was 6.5%. CRP was measured with an automated chemiluminescent immunoassay using the IMMULITE® analyser from DPC (Los Angeles, CA, USA). The CV for CRP was 3.8%. All samples were thawed in a 37°C water bath and analysed directly afterward. Samples from cases and their matched referents were analysed together in a random order. Measurements were made in 2005 by laboratory staff unaware of each participant’s disease status. Storage time has been shown to have a negligible impact on laboratory measurements of frozen samples [30].

### Statistical analysis

Data are presented as proportions, means, and standard deviations (SDs) or median and interquartile range for cases and referents. Significance was tested using the independent sample *t*-test for normally distributed continuous variables, Mann–Whitney test for non-normally distributed continuous variables, and chi-squared for categorical variables.

Conditional logistic regression was used to calculate age- and sex-matched odds ratios (ORs) comparing the risk of incident T2DM in cases and referents. Natural logarithmic transformation was used for CRP, triglycerides, VWF, tPA, PAI-1, and tPA/PAI-1 measurements because these variables were not normally distributed. The ORs with 95% confidence intervals (CIs) were calculated per 1 SD increment for continuous variables. Adjustments were performed for each haemostatic risk factor and potential confounders in three models with complete data sets: 1) BMI, smoking, family history of diabetes, and physical activity; 2) BMI, smoking, CRP, systolic blood pressure, and triglycerides; and 3) model 2 plus FPG and 2hPG levels.

To investigate whether haemostatic factors deteriorate prior to the increase in plasma glucose, cases were divided into two groups: 59 cases with normal glucose values (FPG <6.1 mmol/L and 2hPG <8.9 mmol/L at baseline) and their matched referents, and 98 cases with elevated glucose values (FPG = 6.1-6.9 mmol/L and/or 2hPG = 8.9-12.1 mmol/L at baseline) and their matched referents. Multivariate conditional logistic regression was performed for each fibrinolytic factor, BMI, family history of diabetes, physical activity, smoking, CRP, systolic blood pressure, and triglycerides. ORs with 95% CIs were calculated per 1 SD increment for continuous variables.

Predictive Analytics Software (PASW®) version 18.0 was used for statistical analysis. *P*- <0.05 (two-sided) was considered significant.

### Ethical considerations

This study protocol was approved by the Research Ethics Committee of Umeå University. All participants provided informed consent.

## Results

Cases with T2DM were diagnosed a median 5.5 years (range 0.1-10.6 years) after the baseline health examination in VIP. The baseline characteristics for cases and referents are given in Table 1. Cases reported having a parent or sibling with diabetes more often than referents. No difference was found in smoking status and physical activity at baseline. Hypertension was more common among cases. Cases with incident T2DM had higher baseline systolic and diastolic blood pressure, FPG, 2hPG, BMI, and triglyceride, CRP, tPA, PAI-1, tPA/PAI-1, and VWF levels compared to referents.

**Table 1:** Baseline characteristics for cases with incident type 2 diabetes mellitus and referents

	**Total population**				
	**n**	**Cases**	**n**	**Referents**	**p-value**
Time to T2DM diagnosis, years	157	5.5 ± 2.7	-	-	-
Age, years	157	50.5 ± 8.1	277	50.2 ± 8.3	matched
Sex, male (%)	157	56.7	277	56.7	matched
Family history of T2DM, n (%)	154	48 (31.4)	269	44 (16.4)	< 0.001
Non, Ex, Daily smoking, n (%)	156	56 (35.9), 56 (35.9), 44 (28.2)	272	112 (41.2) 105 (38.6) 55 (20.2)	0.163
Sedentary, Moderate, Active, n (%)	157	23 (14.6), 116 (73.9), 18 (11.5)	277	48 (17.3), 190 (68.6), 39 (14.1)	0.507
Hypertension^*^, n (%)	157	104 (66.2)	277	39 (32.1)	< 0.001
Antihypertensive medication, n (%)	157	41 (26.1)	277	16 (5.8)	< 0.001
Systolic blood pressure, mmHg	157	139.2 ± 18.3	276	127.5 ± 16.8	< 0.001
Diastolic blood pressure, mmHg	157	87.0 ± 12.5	276	79.7 ± 10.4	< 0.001
Fasting plasma glucose, mmol/L	157	5.9 ± 0.8	277	5.2 ± 0.7	< 0.001
2-h plasma glucose, mmol/L	157	8.2 ± 2.1	277	6.5 ± 1.7	< 0.001
BMI, kg/m^2^	156	29.5 ± 4.2	277	25.3 ± 3.6	< 0.001
Total cholesterol, mmol/L	157	6.0 ± 1.0	277	5.7 ± 1.0	< 0.001
HDL, mmol/L	157	1.1 ± 0.3	277	1.3 ± 0.3	0.002
Triglycerides, mmol/L	157	1.7 (1.3 - 2.5)	277	1.1 (0.9 - 1.6)	< 0.001
tPA, ng/mL	156	11.0 (8.7 - 13.7)	276	7.7 (5.6 - 10.5)	< 0.001
PAI-1, ng/mL	157	35.0 (23.9 - 46.1)	276	21.2 (14.6 - 31.3)	< 0.001
tPA/PAI-1 complex, ng/mL	155	7.9 (5.2 - 0.4)	277	3.8 (2.5 - 5.8)	< 0.001
VWF, %	156	142.9 (119.1 - 178.9)	276	124.0 (96.0 - 159.6)	< 0.001

Values are mean ± SD for continuous, and percent for non-continuous variables. Median and interquartile range presented for variables not displaying normal distribution.

*BMI*: body mass index; *CRP*: C-reative protein; *T2DM*: type 2 diabetes mellitus; *tPA*: tissue plasminogen activator; *PAI-1*: plasminogen activator inhibitor-1; *VWF*: von Willebrand factor; *SD*: standard deviation.

The univariate conditional logistic regression analysis showed a significantly increased risk of incident T2DM for family history of diabetes, hypertension, systolic and diastolic blood pressure, FPG, 2hPG, BMI, triglycerides, CRP, tPA, PAI-1, tPA/PAI-1 complex, and VWF (Table 2). The exclusion of patients taking antihypertensive medication did not affect the association of haemostatic variables with incident T2DM (data not shown).

**Table 2:** Risk of incident type 2 diabetes in 157 cases compared to 277 referents

	**OR**	**95% CI**	**p-value**
Family history of T2DM	2.46	1.52 - 3.97	< 0.001
Smoking (daily vs non-smoker)	1.69	0.98 - 2.89	0.059
Physical activity (sedentary vs active)	0.94	0.44 - 2.02	0.878
Hypertension*	3.85	2.48 - 5.97	< 0.001
Systolic blood pressure	2.00	1.58 - 2.54	< 0.001
Diastolic blood pressure	1.93	1.51 - 2.47	< 0.001
Fasting plasma glucose	3.77	2.68 - 5.32	< 0.001
2-h plasma glucose	2.65	2.03 - 3.47	< 0.001
BMI	3.09	2.29 - 4.18	< 0.001
Triglycerides	2.38	1.84 - 3.07	< 0.001
CRP	1.91	1.51 - 2.42	< 0.001
tPA	2.63	1.96 - 3.54	< 0.001
PAI-1	2.33	1.79 - 3.03	< 0.001
tPA/PAI-1 complex	4.02	2.84 - 5.69	< 0.001
VWF	1.73	1.36 - 2.19	< 0.001

* Blood pressure ≥ 140/90 and/or on antihypertensive medication.

Conditional logistic regression analysis. OR and 95% CI, per 1 SD increment.

Referents were matched for age and sex. Natural logarithm transformation was used for haemostatic markers, C-reactive protein and triglycerides.

*OR*: odds ratio; *CI*: confidence interval; *SD*: standard deviation; *BMI*: body mass index; *CRP*: C-reactive protein; *T2DM*: type 2 diabetes mellitus; *tPA*: tissue plasminogen activator; *PAI-1*: plasminogen activator inhibitor-1; *VWF*: vn Willebrand factor.

Multivariate regression analysis was performed for each haemostatic factor (Table 3). In model 1, tPA (OR = 1.79, 95% CI 1.27-2.53), PAI-1 (OR = 1.86, 95% CI 1.34-2.59), tPA/PAI-1 complex (OR = 2.92, 95% CI 1.92-4.43), and VWF (OR = 1.43, 95% CI 1.04-1.94) were independently associated with incident T2DM. In model 2, tPA, PAI-1, and tPA/PAI-1 complex were related to incident T2DM, but VWF (OR =1.33, 95% CI 0.95-1.85) was no significantly associated with incident T2DM. In model 3, only PAI-1 (OR = 1.60, 95% CI 1.03-2.47) and tPA/PAI-1 (OR = 1.70, 95% CI 1.02-2.82) remained independently associated with incident T2DM.

**Table 3:** Risk of incident type 2 diabetes mellitus in relation to each haemostatic variable

		**Crude OR**	Multivariate model 1^*^	**Multivariate model 2**^**†**^	**Multivariate model 3**^**‡**^
	**n cases/n referents**	**OR (95% CI)**	**OR (95% CI)**	**OR (95% CI)**	**OR (95% CI)**
tPA	153/260	2.78	1.79	1.54	1.22
		(2.04 - 3.79)^§^	(1.27 - 2.53)^§^	(1.06 - 2.23)^§^	(0.77 - 1.94)
PAI-1	152/260	2.35	1.86	1.61	1.60
		(1.79 - 3.09)^§^	(1.34 - 2.59)^§^	(1.14 - 2.28)^§^	(1.03 - 2.47)^§^
tPA/PAI-1 complex	154/262	4.05	2.92	2.45	1.70
		(2.83 - 5.79)^§^	(1.92 - 4.43)^§^	(1.56 - 3.84)^§^	(1.02 - 2.82)^§^
VWF	153/261	1.78	1.43	1.33	1.33
		(1.39 - 2.29)^§^	(1.04 - 1.94)^§^	(0.95 - 1.85)	(0.88 - 2.02)

Conditional logistic regression analysis performed with complete data sets. ORs with 95% CIs, per 1 SD increment.

* Model 1: Adjusted for body mass index, smoking (non/ex/daily smoking), family history of T2DM and physical activity (sedentary/moderate/active).

^†^ Model 2: Adjusted for body mass index, smoking (non/ex/daily smoking), family history of T2DM, physical activity (sedentary/moderate/active), C-reactive protein, systolic blood pressure and triglycerides.

^‡^ Model 3: Adjusted for body mass index, smoking (non/ex/daily smoking), family history of T2DM, physical activity (sedentary/moderate/active), C-reactive protein, systolic blood pressure and triglycerides, fasting glucose level and 2 hour capillary glucose level.

^§^ p < 0.05.

Natural logarithm transformation was used for C-reactive protein, triglycerides, tPA, PAI-1, tPA/PAI-1 complex and VWF.

*OR*: odds ratio; *CI*: confidence interval; *SD*: standard deviation; *T2DM*: type 2 diabetes mellitus; *tPA*: tissue plasminogen activator; *PAI-1*: plasminogen activator inhibitor-1; *VWF*: von Willebrand factor.

### Subgroup analysis

**Figure 2:**
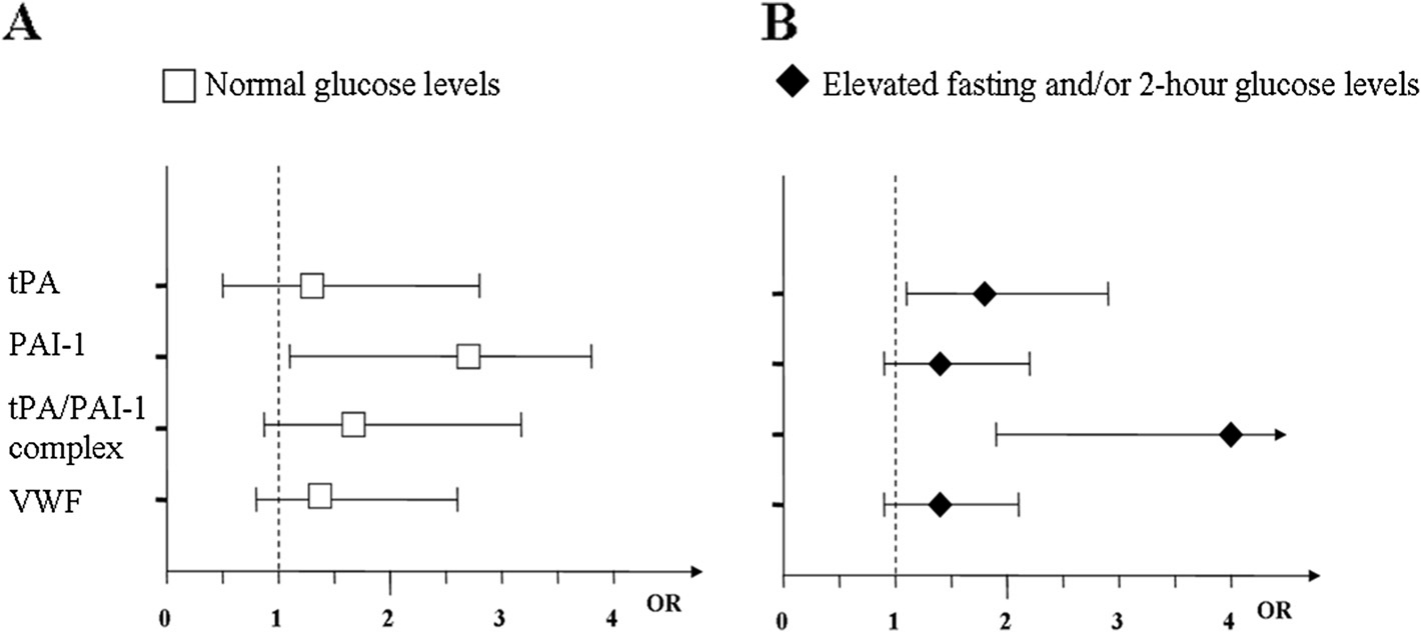
**Forest plots illustrating risk for incident type 2 diabetes in A) cases with normal glucose levels**^**† **^**and B) cases with elevated glucose levels**^**‡**^**.** †Fasting plasma glucose (FPG) < 6.1 and 2-hour capillary plasma glucose (2hPG) < 8.9 mmol/L. ‡ FPG 6.1-6.9 and/or 2hPG 8.9-12.1 mmol/L. Conditional logistic regression analysis performed with complete data sets. ORs with 95% CIs, per increment of 1 SD. Multivariate analysis including body mass index, smoking (non/ ex/ daily smoking), family history of T2DM, physical activity (sedentary/ moderate/ active), C-reactive protein, systolic blood pressure and triglycerides and one of the haemostatic variables (tPA: tissue plasminogen activator; PAI-1: plasminogen activator inhibitor-1, tPA/PAI-1 complex) or VWF: von Willebrand Factor. Natural logarithm transformation was used for C-reactive protein, triglycerides, tPA, PAI-1 and tPA/PAI-1 complex. OR: odds ratio; CI: confidence Interval; SD: standard deviation.

Conditional univariate and multivariate analysis was performed separately for the subgroups of participants with normal glucose levels and elevated glucose levels at baseline (Table 4). Multivariate analysis was carried out with adjustments for BMI, smoking, family history of diabetes, physical activity, CRP, systolic blood pressure, triglycerides, and each haemostatic variable (Figure 2). As the subgroups were based on normal or elevated glucose levels, no adjustments were made for glucose levels.

**Table 4:** Risk of incident type 2 diabetes mellitus for cases with normal glucose levels^*^ and cases with elevated glucose levels^**†**^

		Cases with normal glucose levels*			**Cases with elevated glucose levels**^**†**^	
		**Crude OR**	**Multivariate model‡**		**Crude OR**	**Multivariate model‡**
	**n cases/n referents**	**OR (95% CI)**	**OR (95% CI)**	**n cases/n referents**	**OR (95% CI)**	**OR (95% CI)**
tPA	59/103	2.43	1.24	94/157	3.05	1.82
		(1.50 - 3.91)^§^	(0.57 - 2.70)		(2.02 - 4.61)^§^	(1.11 - 2.98)^§^
PAI-1	57/100	2.66	2.06	95/160	2.20	1.40
		(1.66 - 4.25)^§^	(1.10 - 3.86)^§^		(1.57 - 3.07)^§^	(0.89 - 2.20)
tPA/PAI-1 complex	59/103	3.23	1.67	95/159	4.80	4.01
		(1.93 - 5.41)^§^	(0.87 - 3.22)		(2.90-7.92)^§^	(1.90 - 8.46)^§^
VWF	58/102	1.66	1.43	95/159	1.86	1.35
		(1.13 - 2.46)^§^	(0.79 - 2.59)		(1.35 - 2.58)^§^	(0.87 - 2.09)

* Fasting plasma glucose (FPG) < 6.1 and 2-hour capillary plasma glucose (2hPG) < 8.9 mmol/L.

^†^ FPG 6.1-6.9 and/or 2hPG 8.9-12.1 mmol/L.

Conditional logistic regression analysis performed with complete data sets. ORs with 95% CIs, per 1 SD increment.

^‡^ Model including body mass index, smoking (non/ex/daily smoking), family history of T2DM, physical activity (sedentary/moderate/active), C-reactive protein, systolic blood pressure and triglycerides and one of the fibrinolytic variables (tPA: tissue plasminogen activator; PAI-1: plasminogen activator inhibitor-1 or tPA/PAI-1 complex).

^§^ p < 0.05.

Natural logarithm transformation was used for C-reactive protein, triglycerides, tPA, PAI-1 and tPA/PAI-1 complex.

*OR*: odds ratio; *CI:* confidence interval; *SD*: standard deviation.

For participants with normal glucose levels, all haemostatic variables were significantly associated with incident diabetes in the univariate analysis (tPA: OR = 2.43, 95% CI 1.50-3.91; PAI-1: OR=2.66, 95% CI 1.66-4.25; tPA/PAI-1 complex: OR=3.23, 95% CI 1.93-5.41; VWF: OR=1.66, 95% CI 1.13-2.46). In multivariate analysis, only PAI-1 remained significant (OR=2.06, 95% CI 1.10-3.86). For participants with elevated glucose levels, all haemostatic variables were significantly associated with incident diabetes in the univariate analysis (tPA: OR=3.05, 95% CI 2.02-4.61; PAI-1: OR=2.20, 95% CI 1.57-3.07; tPA/PAI-1 complex: OR=4.80, 95% CI 2.90-7.92; VWF: OR=1.86, 95% CI 1.35-2.58). After adjustments, tPA (OR=1.82, 95% CI 1.11-2.98) and tPA/PAI-1 complex (OR=4.01, 95% CI 1.90-8.46) remained significant.

## Discussion

This study shows that elevated levels of tPA, PAI-1, and tPA/PAI-1 complex precede incident diabetes after adjusting for metabolic and cardiovascular risk factors. The association of PAI-1 with incident T2DM was also detected in participants with normal blood glucose levels at baseline, indicating that fibrinolytic impairment occurs prior to increased glucose levels.

An earlier study of individuals with normal OGTT who were followed for 9 years revealed that the 15 participants who developed T2DM had significantly higher tPA antigen levels compared to referents after adjusting for factors associated with metabolic syndrome [12]. In this larger study, we found an association between tPA and incident T2DM after adjusting for metabolic and cardiovascular risk factors. This association disappeared after additional adjustments were made for FPG and 2hPG. Diabetic patients have active coagulation and hypofibrinolysis induced by both chronic and acute hyperglycaemia [6,7]. An experimental investigation has shown that high glucose levels increase the production of tPA antigen from human mesengial cells [31], suggesting that the tPA increase found in this study may be stimulated by glucose.

Both PAI-1 antigen and the tPA/PAI-1 complex were significantly associated with incident T2DM in all three multivariate models. This result is in agreement with other reports of a relationship between PAI-1 and incident diabetes [18,19] independent of metabolic syndrome [32]. Visceral adipose tissue has been shown to increase PAI-1 secretion [33]. In a cross-sectional study, both tPA and PAI-1 activities and antigens were associated with metabolic syndrome parameters in patients with or without T2DM [34]. However, the association found in this study was independent of BMI and other components of the metabolic syndrome.

In participants with normal glucose levels, only PAI-1 remained significantly associated with incident diabetes in the multivariate analysis. In participants with elevated glucose levels, only tPA and tPA/PAI-1 complex remained significantly associated with incident diabetes. This finding implies that tPA and PAI-1 play different roles in the pathophysiology of T2DM, with PAI-1 levels increasing early in the disease. As pre-diabetic changes develop and blood glucose levels start to increase, tPA and tPA/PAI-1 complex increase.

The influence of PAI-1 on the development of T2DM was discussed previously in a variety of contexts. Obesity has been shown to be associated with chronic inflammation [35]. CRP has been shown to up-regulate the gene expression of PAI-1 in human aortic endothelial cells [36]. Resident macrophages in the adipose tissue produce cytokines, such as tumour growth factor (TGF)-β and tumour necrosis factor (TNF)-α, that also up-regulate PAI-1 [37,38], indicating that the increase in PAI-1 occurs parallel to the obesity-induced development of T2DM. Therefore, the up-regulation of PAI-1 early in the development of T2DM may be seen as a result of the metabolic syndrome. Mouse models have raised the possibility that PAI-1 is not merely a product of obesity, but has a causal role in obesity and insulin resistance, as PAI-1 inhibition has the potential to reduce obesity and improve insulin sensitivity [39,40], possibly via PAI-1 functioning as an integrator of cell signalling [41]. In T2DM patients, PAI-1 antigen levels have been shown to decrease following metformin treatment [42]. Weight reduction in obese individuals was also associated with a decrease in PAI-1 antigen levels [43], which is explained, in part, by the loss of PAI-1-secreting visceral adipose tissue [33] but may also be affected by other PAI-1 functions.

A previous investigation reported no association between VWF and incident T2DM after adjusting for IL-6, adiponectin, and γ-glutamyl transferase [13]. In our study, VWF was associated with T2DM in the univariate analysis and model 1, but this association was lost after further adjustments in model 2 for CRP, systolic blood pressure, and triglycerides. This result is in line with previous research showing that inflammatory markers can activate endothelial cells that produce VWF [24].

### Strengths and limitations

The major advantage of this study is the prospective nested case-referent study design within a well-defined population-based intervention programme, the VIP. Men and women age 30 to 60 years underwent an extensive baseline health examination regarding cardiovascular risk factors at their local health centre. Diabetic patients were defined by FPG and OGTT. Participants who developed T2DM were examined years before the manifest diagnosis.

The number of missing cases is high, as 80 of the original 237 cases did not have blood samples available for laboratory analysis of the haemostatic variables. Baseline data for 60 cases without blood samples available showed that no significant difference existed in diastolic and systolic blood pressure, FPG levels, or BMI compared to the cases in the study. As data on the cases without blood samples was similar to the data on those included in the study, selection bias is unlikely. Lifestyle changes recommended in the initial health examination, such as weight loss and smoking cessation, may have influenced the results. In a 10-year follow-up study [44], VIP participants with baseline glucose intolerance or T2DM were the most likely to avoid weight gain, but this occurrence most likely affected the incidence of T2DM and not the relationship between baseline fibrinolytic data and the risk of developing diabetes in a given individual. The vast majority of participants were Caucasian, limiting the possibility of generalising the results to other ethnicities. A risk of regression dilution bias exists because only a single baseline blood sample was used, which may decrease the estimations of the observed associations.

## Conclusions

Elevated levels of fibrinolytic variables precede the manifestation of T2DM after adjusting for metabolic and cardiovascular risk factors and can be detected several years before changes in glucose tolerance

## Abbreviations

2hPG: 2-hour plasma glucose after oral glucose tolerance test; BMI: Body mass index; CRP: C-reactive protein; FPG: Fasting plasma glucose; IL-6: Interleukin-6; OGTT: Oral glucose tolerance test; PAI-1: Plasminogen activator inhibitor-1; SD: Standard deviation; T2DM: Type 2 diabetes mellitus; tPA: Tissue plasminogen activator; TGF- β: Tissue growth factor- β; TNF-α: Tumour necrosis factor- α; VIP: Västerbotten Intervention Programme; VWF: Von Willebrand factor.

## Competing interests

The authors have no conflict of interest to disclose.

## Authors’ contributions

JHB: Data collection, analysis and interpretation of the results, drafting and revision of the manuscript. MN: Conception and design of the study, data collection, drafting and revision of the manuscript. JHJ: Data collection, analysis and interpretation of the results, drafting and revision of the manuscript. ME: Data collection, drafting and revision of the manuscript. JWE: Conception and design of the study, data collection, revision of the manuscript. BL: Conception and design of the study and revision of the manuscript. LJ: Data collection, analysis and interpretation of the results, drafting and revision of the manuscript. All authors read and approved the final manuscript.
